# Mechanisms of non-coding RNA-modulated alternative splicing in cancer

**DOI:** 10.1080/15476286.2022.2062846

**Published:** 2022-04-15

**Authors:** Xiaolin Wang, Jinghan Hua, Jingxin Li, Jiahui Zhang, Emmanuel Enoch Dzakah, Guozhen Cao, Wenchu Lin

**Affiliations:** a High Magnetic Field Laboratory, Hefei Institutes of Physical Science (Hips), Chinese Academy of Sciences, Hefei, Anhui, P. R. China; b University of Science and Technology of China, Hefei, Anhui, P. R. China; c Key Laboratory of High Magnetic Field and Ion Beam Physical Biology, HIPS, Chinese Academy of Sciences, Hefei, Anhui, P. R. China; d High Magnetic Field Laboratory of Anhui Province, Hefei, Anhui, P. R. China; e Department of Molecular Biology and Biotechnology, School of Biological Sciences, College of Agriculture and Natural Sciences, University of Cape Coast, Cape Coast, Ghana

**Keywords:** Alternative splicing, splicing factor, cancer progression, miRNA, lncRNA, circRNA

## Abstract

Alternative splicing (AS) is a common and pivotal process for eukaryotic gene expression regulation, which enables a precursor RNA to produce multiple transcript variants with diverse cellular functions. Aberrant AS represents a hallmark of cancer, engaged in all stages of tumorigenesis from initiation to metastasis. Accumulating pieces of evidence have revealed the involvement of non-coding RNAs (ncRNAs) in regulating AS in human cancers. In this review, we overview the underlying mechanisms of non-coding RNAs, including microRNAs (miRNAs), long non-coding RNAs (lncRNAs) and circular RNAs (circRNAs) modulated AS at diverse levels in human cancers, and summarize their regulatory functions in tumorigenesis.

## Introduction

Over 90% of human intron-containing genes undergo alternative splicing (AS) to form multiple mRNA isoforms [1–3]. AS is generated via splicing at different splice sites or selective removal of introns from a single precursor mRNA (pre-mRNA) [2]. The process is highly and tightly controlled, mainly relying on splicing sites and splicing factors (SFs) such as serine/arginine-rich (SR) proteins, heterogeneous nuclear ribonucleoproteins (hnRNPs), and tissue-specific SFs [4–6]. In addition, transcriptional repression or activation, chromatin structure and histone modification are also implicated in AS regulation [7,8]. The most prevalent types of AS are the inclusion or skipping of an entire exon (cassette exon), mutually exclusive exon, the selection for the alternative 5’ or 3’ splice site (SS) of exons, and intron retention [6,7].

A series of evidence has demonstrated that aberrant AS is engaged in multiple biological processes, including cancer initiation, development and metastasis [9–13]. For instance, myeloid cell leukaemia-1 (MCL1) and BCL2 like 1 (BCL-x) experience AS to produce either the long anti-apoptotic variants (MCL1L and BCL-xL) or the short pro-apoptotic isoforms (MCL1S and BCL-xS) [7]. AS of MCL1 and BCL-x is orchestrated by SFs, including SF3B1 and SRSF1 [14,15]. The increased expressions of MCL1L and BCL-xL facilitate cancer progression and are associated with resistance to diverse chemotherapeutic agents in multiple cancer types [7,15]. In addition, the vascular endothelial growth factor-A (VEGF-A) gene undergoes AS events, which could generate two isoforms, VEGF-165 and VEGF-165b [7]. It is well known that angiogenesis is an essential initial step in cancer progression [16]. The switch from anti-angiogenic VEGF-165b to pro-angiogenic VEGF-165 stimulates angiogenesis required for tumour growth and progression [7]. Intriguingly, analyses of more than 8,000 tumours across 32 cancer types revealed thousands of splicing variants in cancers but not non-malignant tissues [17]. These results enable AS to contribute to almost every hallmark of cancer progression and exhibit prognostic values [17–19].

The majority of the human genome is actively transcribed into a diverse group of non-coding RNAs (ncRNAs) that are not translated into proteins [20,21]. NcRNAs have emerged as key regulators of tumorigenesis [22,23]. Increasing literature has shown that many aberrantly expressed splicing variants in cancer are directly regulated by ncRNAs [24–27]. A comprehensive overview of the roles of ncRNAs in regulating AS in human cancers will deeply expand our understanding of their underlying mechanisms in tumorigenesis, which provides new insights to develop targeted cancer treatment strategies. In the present review, we discuss the available evidence on mechanisms of AS modulated by microRNAs (miRNAs), long non-coding RNAs (lncRNAs) and circular RNAs (circRNAs) in cancer and discuss the potential and clinical application values of manipulating AS in cancer therapy.

## Regulation of alternative splicing by miRNAs

MicroRNAs (miRNAs) are small non-coding RNA molecules that are approximately 22 nucleotides in length with high conservation across species [28,29]. MiRNAs exert a considerable influence on the modulation of gene expression, mainly by incorporating into the RNA-induced silencing complex (RISC) to the 3’ untranslated regions (3’ UTRs) of genes [28]. Moreover, several miRNAs have been reported to exert their functions via binding to their targets’ 5’ UTRs or coding regions [30,31]. Functional roles of miRNAs in cancer have been reviewed elsewhere [29]; herein, we summarize the mechanisms of miRNA-mediated alternative splicing in cancer progression.

**Figure 1.**
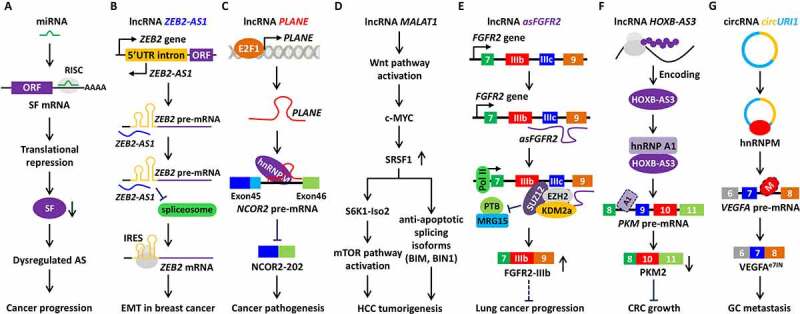
Mechanisms of ncRNA-mediated AS in human cancers. **A.** MiRNAs are mainly incorporated into the RNA-induced silencing complex (RISC) to the 3’ UTRs of SFs, leading to translational repression of SFs. The decreased SFs contribute to dysregulated AS in cancer. UTR, untranslated region. **B**. The lncRNA ZEB2-AS1 transcribed from the antisense strand of the ZEB2 locus, forms an RNA-RNA duplex encompassing the 5’ splice site of the 5’ UTR intron within the ZEB2 pre-mRNA *in cis*. The dsRNA blocks the binding of the spliceosome resulting in intron retention. An internal ribosome entry site (IRES) proximal to the ZEB2 start code (AUG), is retained in the intron and favours ZEB2 translation, contributing to the EMT in breast cancer. EMT, epithelial-mesenchymal transition. **C**. The lncRNA PLANE is increased in diverse cancers, driven by E2F1-mediated transcriptional activation. PLANE interacts with the intron 45 of NCOR2 pre-mRNA via RNA-RNA base pairing *in trans* to facilitate the binding of hnRNPM, leading to the repression of the NCOR2-202 transcript and the promotion of tumorigenesis. **D**. The lncRNA MALAT1 is upregulated in HCC and promotes HCC tumorigenesis through transcriptional induction of the oncogenic SRSF1, which is activated by the Wnt pathway/c-MYC axis. MALAT1-mediated upregulation of SRSF1 results in the modulation of AS, including the production of anti-apoptotic splicing isoforms (such as BIM and BIN1) and the oncogenic splicing isoform S6K1-Iso2 to activate the mTOR pathway. HCC, hepatocellular carcinoma. **E**. The lncRNA asFGFR2 recruits the PRC2 proteins (EZH2 and SUZ12) and the H3K36 demethylase KDM2a to the parental locus, which impairs the binding of the chromatin-splicing adaptor complex MRG15–PTB to the exon IIIb, eventually generating the FGFR2-IIIb isoform. **F**. The lncRNA HOXB-AS3 encodes the HOXB-AS3 peptide, which prevents hnRNPA1 binding to PKM pre-mRNA flanking exon 9, resulting in the decrease of the PKM2 isoform and the suppression in CRC growth. CRC, colorectal cancer. **G**. The circRNA circURI1 is highly expressed in GC and sequesters hnRNPM protein in a sequence-dependent manner to modulate AS of migration-related genes (e.g. VEGFA), consequently inhibiting GC metastasis. GC, gastric cancer; VEGFA^e7IN^, exon 7 inclusion of VEGFA.

MiRNAs modulate AS by targeting SFs or RNA-binding proteins (RBPs) in cancer [26,31] (Fig. 1A). For instance, in hepatocellular carcinoma (HCC), miR-133b represses the translation of SF3B4 mRNA to disrupt SF3B4-regulated AS, inhibiting cell proliferation and metastasis [32]. MiR-200c and miR-375 exhibit the translational repression of Quaking (QKI), a well-characterized RBP, to affect QKI-mediated AS, thereby influencing cancer-associated epithelial cell plasticity [26]. Additionally, Yang and his colleagues have demonstrated that the miR-212/hnRNPH1 axis impacts prostate tumorigenesis through downregulation of the expressions of androgen receptor (AR) and its splice variant AR3 [33].

MiRNAs can also function by specifically targeting alternatively spliced transcripts [34]. For example, Krüppel like factor 6 (KLF6) undergoes AS to generate two antagonistic isoforms [34]. The full-length KLF6 (KLF6-FL) is a tumour-suppressive isoform, while KLF6 splice variant 1 (KLF6-SV1) possesses an oncogenic effect in liver cancer [34]. Alternative 5’ splice sites located within exon 2 lead to the generation of KLF6-SV1, which lacks a 154-bp-coding region. MiR-1301 could enhance liver cancer cell migration and angiogenesis by targeting the 154-bp sequence within KLF6-FL rather than KLF6-SV1 [34]. Although miRNAs targeting alternatively spliced exons are rarely reported, it is no doubt that more miRNAs acting in this manner will be identified and characterized in the future.

## Roles of lncRNAs in regulating AS

Long non-coding RNAs (lncRNAs) are an abundant class of endogenous RNA molecules with more than 200 nucleotides [21,35]. LncRNAs are generally transcribed from RNA polymerase II (Pol II) with poly (A) tails and 5’ caps, while there are still several non-Pol II-transcribed and non-poly-adenylated lncRNAs [21,35,36]. LncRNAs predominantly localize to the nucleus, and exhibit lower expression levels and poorer conservation than message RNAs (mRNAs) [21,35]. LncRNAs have emerged as indispensable regulators in various biological processes, including chromatin modification and transcriptional regulation, and numerous human diseases like diabetes mellitus and cancers [37,38]. Accumulating evidence has demonstrated that lncRNAs exert critical roles in cancer progression via regulating AS in the past few decades [25,27].

### RNA-RNA interaction

LncRNAs interact with pre-mRNAs via complementary sequences [27,39]. The lncRNA-pre-mRNA interactions result in the selection of splicing sites and the recruitment of SFs, eventually modulating the AS of targeted pre-mRNAs [27].

Antisense lncRNAs always bind to the parental pre-mRNAs to regulate AS *in cis* [38,40]. For instance, the lncRNA ZEB2-AS1 (ZEB2 antisense RNA 1) blocks the recognition of the spliceosome in the splicing sites of the ZEB2 pre-mRNA via RNA-RNA interaction [41] (Fig. 1B). The block results in intron retention at the ZEB 5’ untranslated region (UTR) [41]. This intron contains an internal ribosome entry site (IRES), bound by ribosomes to promote IRES-mediated ZEB2 translation, consequently activating epithelial-mesenchymal transition (EMT) and facilitating metastasis in breast cancer [41]. Another example with the *cis*-regulatory role in modulating AS is the lncRNA EGOT (Eosinophil granule ontogeny transcript) [42]. EGOT is an antisense intronic lncRNA transcribed from the genomic region of ITPR1 [42]. Mechanistically, EGOT directly binds to ITPR1 pre-mRNA to form a pre-ITPR1/EGOT dsRNA and recruits hnRNPH1 to promote pre-ITPR1 AS [42]. EGOT-triggered pre-ITPR1 AS contributes to the expression of the ITPR1 protein, which sensitizes cells to paclitaxel in cancer therapy [42].

Some lncRNAs exert the function of modulating AS *in trans* via RNA-RNA interaction [27,43,44]. For example, the lncRNA PLANE (Pan-cancer lncRNA activating NCOR2 responsive to E2F1) is generally upregulated in multiple cancer types via genomic amplification and E2F1-mediated transcriptional activation [45] (Fig. 1C). The effect of PLANE on cancer proliferation and tumorigenicity is associated with a tumour-suppressive NCOR2 AS isoform (NCOR2-202) [45]. Mechanistically, PLANE interacts with NCOR2 to facilitate the binding of hnRNPM within intron 45 of the NCOR2 pre-mRNA, eventually repressing the production of NCOR2-202 [45]. Similarly, the lncRNA CCAT2 (Colon cancer-associated transcript 2) directly binds to intron 14 of glutaminase (GLS) pre-mRNA and enhances the recruitment of the cleavage factor I (CFIm) complex [46]. In addition, the CCAT2-pre-GLS-CFIm interaction fine-tunes the GLS AS by selecting the poly (A) site to promote the expression level of glutaminase C (GAC) splicing variant [46]. CCAT2-induced GAC splicing variant contributes to cell proliferation and metastasis in colorectal cancer (CRC) [46].

Collectively, lncRNAs can modulate AS to exert distinct functions in cancers via RNA-RNA interaction *in cis* or *trans*.

### Interacting with RNA binding proteins

LncRNAs can diametrically modulate AS by interacting with RBPs to regulate their targeted genes [27,47,48]. For instance, the lncRNA DGCR5 (DiGeorge syndrome critical region gene 5) is highly expressed in oesophageal squamous cell carcinoma (ESCC) and is associated with poor prognosis in patients with ESCC [47]. Functional studies have demonstrated that DGCR5 silencing significantly suppresses ESCC cell proliferation, migration and invasion *in vitro* [47]. Mechanistically, DGCR5 stabilizes SRSF1 by directly binding to SRSF1, and thus stimulates AS events such as producing the long anti-apoptotic MCL1L variant [47]. In addition, the lncRNA LASTR (LncRNA associated SART3 regulation of splicing) controls splicing efficiency by regulating the LASTR-interacting partner SART3, a recycling factor of the splicing machinery [48]. LASTR promotes the dissociation of SART3 from a transient SART3-U4/U6 small nuclear ribonucleoproteins (snRNP) complex, ultimately increasing the fitness of breast cancer cells [48].

### Transcriptional regulation

AS is often coupled with transcription and lncRNAs could also modulate AS by transcriptional regulation [7,49–51]. For example, the lncRNA Pvt1b suppresses the transcriptional activity and level of c-Myc, resulting in the repression of lung cancer proliferation [49]. The oncogenic transcription factor c-Myc is known to activate the transcription of hnRNP proteins to deregulate pyruvate kinase mRNA splicing in cancer [52]. Additionally, the lncRNA MALAT1 (Metastasis-associated lung adenocarcinoma transcript 1), is up-regulated and exerts oncogenic activity in hepatocellular carcinoma (HCC) [51]. Mechanistic investigation revealed that MALAT1 transcriptionally activates the expression of the oncogenic splicing factor SRSF1 via targeting the Wnt pathway/c-Myc axis [51]. Further evidence implies that MALAT1-induced SRSF1 triggers an AS program to facilitate the production of anti-apoptotic splicing isoforms and the oncogenic splicing isoform (S6K1-Iso2) [51] (Fig. 1D). In addition, MALAT1 interacts with a specific set of SRSFs including SRSF1, to impair the distribution of SFs and modulate SRSF phosphorylation [53]. MALAT1 can also function as an SF decoy to modulate B-MYB AS, contributing to cell cycle progression [54].

### Chromatin remodelling

LncRNAs have been shown to participate in AS mediated by chromatin structure and histone modifications [55–57]. For instance, a highly conserved antisense lncRNA called *asFGFR2*, derived from the human FGFR2 locus, preferentially resides in the nucleus [56]. *asFGFR2* enhances epithelial-specific AS of FGFR2 through recruitment of chromatin modifiers including polycomb repressive complex 2 (PRC2) and the histone demethylase KDM2a to the FGFR2 genomic region [56] (Fig. 1E). Therefore, it establishes a unique chromatin environment that disrupts the binding of the chromatin-adaptor complex MRG15-PTB [56]. The environment thus favours the inclusion of the alternatively spliced exon IIIb and consequently produces the FGFR2-IIIb isoform, which participates in the lung cancer progression [56]. The lncRNA OIP-AS1 (OIP5 antisense RNA 1) also known as Cyrano, is retained in the nucleus and directly interacts with numerous nuclear proteins including SMARCA4 [57]. The OIP-AS1-interacting protein, SMARCA4 is a component of the SWI/SNF chromatin-remodelling complex, which regulates the expression of the OIP5 oncogene via binding to its promoter [57].

### Generating functional polypeptide

Even though the vast majority of lncRNAs are thought to be non-coding, a small subset of lncRNAs exhibits the translational effects to encode small peptides under certain circumstances [58–60]. For example, the putative lncRNA HOXB-AS3 gives rise to a conserved and functional 53-amino acid peptide, whose low level correlates with a poor prognosis in CRC patients [60] (Fig. 1F). *In vitro* and *in vivo* observations reveal that the HOXB-AS3 peptide represses CRC growth [60]. Mechanistic studies have revealed that the HOXB-AS3 peptide interacts with the arginine residues in the RNA-binding RGG box of hnRNPA1 [60]. The HOXB-AS3 peptide sequesters hnRNPA1 to modulate AS of pyruvate kinase M (PKM) via blocking the hnRNPA1ʹs binding to the flanking region of PKM exon 9 [60]. Modulation of PKM AS leads to the decrease in the formation of PKM2 isoform, eventually contributing to the repression of CRC growth [60]. In addition, lncRNA LOC90024 promotes CRC tumorigenesis and progression by encoding a small peptide named Splicing Regulatory Small Protein (SRSP) [61]. Mechanistically, SRSP interacts with several splicing regulators, such as SRSF3, to regulate mRNA splicing [61]. SRSP increases the binding of SRSF3 to exon 3 of transcription factor Sp4, resulting in the formation of the cancerous isoform Sp4-L and the inhibition of the non-cancerous isoform Sp4-S [61].

### Splice switch from mRNA to lncRNA

With the deep understanding of gene expression, multiple lines of evidence have pointed out that several bi-functional genes undergo alternative splicing into both mRNAs and lncRNA variants [62–65]. For example, the protein phosphatase 1 regulatory subunit 10 (PPP1R10 also known as PNUTS) gene undergoes AS to produce lncRNA-PNUTS besides protein-coded PNUTS [63]. The AS event is regulated by hnRNP E1, which binds to a BAT structural element located at the alternative splice site in exon 12 of PNUTS pre-mRNA [63]. As a result, the lncRNA-PNUTS is up-regulated during breast cancer progression and regulates tumour implantation, cancer growth and metastasis through the miR-205/ZEB/E-cadherin axis [63]. Similarly, AS of the PD-L1 gene can give rise to the PD-L1 mRNA and the lncRNA transcript (PD-L1-lnc) [64]. The lncRNA PD-L1-lnc facilitates cell proliferation and invasion by directly interacting with c-Myc and enhancing its transcriptional activity in lung adenocarcinoma (LUAD) [64]. In addition, a recent study has reported that the ASCC3 precursor is alternatively spliced into two isoforms, the longer transcript encoding ASCC3 protein and the shorter variant serving as a lncRNA [65]. The two ASCC3 isoforms display antagonistic effects on transcriptional recovery after UV-induced DNA damage [65]. UV irradiation induces a shift from the long protein-coding ASCC3 isoform to the short non-coding ASCC3 transcript [65]. Further study has revealed that the ASCC3 protein functions in the context of the ASCC complex and maintains transcriptional repression in response to DNA damage, whereas ASCC3 lncRNA localizes in the nucleus and is required for transcriptional recovery [65]. Overall, the above studies indicate that lncRNA and mRNA transcripts from this single gene might exert the functional roles dependent or independent of each other.

## CircRNA-modulated alternative splicing in cancer

Circular RNAs (circRNAs) are naturally endogenous covalently closed RNA molecules back-spliced from pre-mRNA or other RNA circularization mechanisms [66,67]. In contrast to linear RNAs, circRNAs are resistant to RNA exonuclease, due to their loop structures, which provide them with promising features to act as potential biomarkers or therapeutic targets [66,67]. In general, reverse complementary sequences such as Alu elements in the flanking region of circularized exons and various RBPs such as hnRNP L, QKI and hnRNPM are responsible for circRNA biogenesis [68–72]. Accumulating evidence has demonstrated that circRNAs play essential and pivotal roles in tumorigenesis through distinct mechanisms including serving as miRNA sponges, interacting with RBPs, transcriptional regulation and acting as templates for translation [66,67,73]. For instance, we have found that circRNA (*circURI1*) could modulate AS to engage in cancer progression and metastasis [24] (Fig. 1G).

The circRNA circURI1 back-spliced from exons 3–4 of URI1 is identified from circRNA profiling of 5 paired gastric cancer (GC) and adjacent non-cancerous (paraGC) specimens [24]. CircURI1 exhibits a higher expression level in GC compared with paraGC tissues and facilitates GC metastasis *in vitro* and *in vivo* [24]. The RBP, hnRNPM is identified as the circURI1-interacting protein and does not regulate circURI1 biogenesis [24]. The Alu elements in the flanking introns contribute to circURI1 circularization [24]. Mechanistic studies have demonstrated that circURI1 behaved as a decoy of hnRNPM in a sequence-dependent manner to modulate AS of a subset of genes related to cell migration, thus suppressing GC metastasis [24]. VEGFA is a functional target of circURI1 and circURI1 can promote exon 7 inclusion of VEGFA (VEGFA^e7IN^) [24]. CircURI1-induced VEGFA^e7IN^ possesses a more remarkable ability to prevent the promoting effect of circURI1 silencing on GC cell invasion than exon 7 exclusion of VEGFA (VEGFA^e7EX^) [24]. This study firstly reported the circRNA-mediated alternative splicing in cancer metastasis, expanding the current knowledge regarding the molecular mechanism of circRNA in cancers.

One of the common mechanisms for circRNAs is to serve as miRNA sponges or competing endogenous RNAs (ceRNAs) [74–78]. Once the targets of miRNAs are SFs, these circRNAs might be associated with AS. For example, circUHRF1 enhances oral squamous cell carcinoma (OSCC) tumorigenesis by modulating the expression of epithelial splicing regulatory protein 1 (ESRP1) through the miR-526b-5p/c-Myc/TGF-β1 axis [79]. Nevertheless, there is no direct evidence to demonstrate that circRNAs modify AS via acting as miRNA sponges to regulate the SF expressions, which might be a new direction for further investigation.

## Conclusions and perspectives

Although ncRNA-mediated AS in physiological and pathological conditions has drawn more attention, the corresponding functional and mechanistic characterization is still in its infancy [7,27]. Up to now, only a few ncRNAs have been identified to engage in AS in cancer [24,26,49]. Considering that aberrant AS contributes to almost every hallmark of cancer and ncRNAs exhibit essential regulatory roles in human diseases, systematic knowledge regarding the involvement of ncRNAs in modulating AS in cancer is helpful for profound understanding the unique characteristics and biological functions of AS and ncRNAs, and the discovery of potential biomarkers and therapeutic targets. In this review, we discuss how the current ncRNAs including miRNAs, lncRNAs and circRNAs modulate AS in cancer progression and metastasis. Interestingly, a single lncRNA such as MALAT1 could regulate AS at multiple levels, while several different lncRNAs can interact with the same SF to modulate AS [47,51,53]. Construction of the lncRNA-SF regulatory network in modulating AS will help decipher the effects of lncRNAs on AS events in the future.

Given that AS events act in cell type-, tissue- and developmental stage-dependent manners, and specific AS contributes to tumorigenesis, manipulating AS appears to be a promising therapeutic strategy for cancer treatment [7,80–83]. Splice-switching oligonucleotides (SSOs), which are specialized antisense oligonucleotides (ASOs) targeting pre-mRNAs, are validated as an effective and practical approach towards modulating AS events [84]. Diverse SSOs have been approved for the treatment of Duchenne muscular dystrophy (DMD) and Spinal muscular atrophy (SMA), among which the most advanced SSOs are now in Phase 3 clinical trials for the treatment of DMD [7,84,85]. The clustered regularly interspaced short palindromic repeats (CRISPR)/Cas9-mediated genomic editing has also been employed to manipulate AS via point mutations in splice sites, small insertions or deletions leading to partial alteration of splicing, or unexpected large deletions removing exons [86,87]. In addition, the artificial RNA with a polypyrimidine tract (Py) site at the 5’ end and a gene-specific antisense sequence at the 3’ end has already been used as an effective biotechnology tool to manipulate the splicing pattern for several genes [43,44]. The artificial RNA functions through the *trans* delivery of SFs such as U2 small nuclear RNA auxiliary factor 2 (U2AF65) to the targeted pre-mRNAs [43]. Although the theoretical evidence for these technologies targeting AS is enough, the clinical tests for the effectiveness of these approaches are still absent in the management of aberrant AS-related human diseases; hence, further exploration is required to validate the feasibility of AS manipulation.

In conclusion, a better understanding of the underlying molecular mechanisms of ncRNA-mediated AS expands the energetic roles of ncRNAs and AS in human diseases.
